# Re-imagining metastatic breast cancer care delivery: a patient-partnered qualitative study

**DOI:** 10.1007/s00520-023-08201-8

**Published:** 2023-11-30

**Authors:** Mya L. Roberson, Anna Henricks, Joshua Woods, Lesley Glenn, Julia Maues, Deltra James, Sonya Reid

**Affiliations:** 1 Gillings School of Global Public Health, Department of Health Policy and Management, University of North Carolina at Chapel Hill, 1106B McGavran Greenberg Hall, 135 Dauer Drive, CB#7411, Chapel Hill, NC 27599-7411 USA; 2 Lineberger Comprehensive Cancer Center, Chapel Hill, NC USA; 3 Vanderbilt University School of Medicine, Nashville, TN USA; 4 Department of Health Policy, Vanderbilt University School of Medicine, Nashville, TN USA; 5 Project Life, Central Point, OR USA; 6 Guiding Researchers and Advocates to Scientific Partnerships (GRASP), Baltimore, MD USA; 7 Department of Medicine, Division of Hematology and Oncology, Vanderbilt University School of Medicine, Nashville, TN USA

**Keywords:** Qualitative, Metastatic breast cancer, Cancer outcomes, Cancer survivorship, Supportive care

## Abstract

**Purpose:**

While significant progress in metastatic breast cancer (MBC) treatment has prolonged survival and improved prognosis, there remain substantial gaps in providing patient-centered supportive care. The specific care delivery needs for metastatic cancer differ from that of early-stage cancer due to the incurable nature and lifelong duration of the condition. The objective of this study was to assess how patients living with MBC would re-imagine cancer care delivery.

**Methods:**

This qualitative study was conducted in partnership with patient-led organizations *Guiding Researchers and Advocates to Scientific Partnerships* (*GRASP*) and *Project Life*, a nonprofit, online wellness community founded by patients with MBC for patients living with MBC. Virtual semi-structured interviews (*n* = 36) were conducted with *Project Life* members purposively sampled from the groups’ overall membership. The interview guide contained items surrounding patients’ lived experiences of MBC, greatest unmet needs related to care, and perspectives on virtual wellness community involvement. Interviews were coded using two-stage deductive and inductive analysis.

**Results:**

Three major themes for re-imagining cancer care delivery were identified, including holistic care, information needs, and conceptual shifts. Within these several subthemes emerged with patients re-imagining referrals to non-oncological services, caregiver support, acceptance of integrative medicine, streamlined clinical trial enrollment, curated quality patient resources, MBC-specific terminology and approaches, long-term life and goal-of-care planning, and patient-centered voice throughout.

**Conclusion:**

People living with metastatic cancers have specific supportive care needs. These findings highlight patient-driven areas for re-imagination that are most salient for individuals with MBC.

## Introduction

Approximately 12% of women in the USA will develop breast cancer in their lifetime, making it the most common cancer diagnosed among women [1, 2]. In part because of treatment advances, the projected prevalence of women living with metastatic breast cancer (MBC) in the USA in 2025 is approximately 170,000 women [3]. Although significant progress MBC treatment has led to increased survival, large gaps in supportive care remain for people with metastatic cancers [4]. New therapeutic options for metastatic cancers have primarily focused on efficacy and effectiveness, with less emphasis on treatment-related sequelae, including reduced quality of life [5].

Many patients with MBC report a lack of knowledge about their diagnosis as well as a lack of support for their caregivers and family members [6]. Individually tailored social support, opportunities for shared decision-making, and early access to palliative care have been shown effective in increasing positive outcomes and patient satisfaction among people living with MBC [7–9]. In addition, despite the importance of clinical trials for therapeutic advances, clinical trial participation among MBC patients is often low due to strict eligibility criteria for entry [10]. Many patients with MBC are simply not aware of clinical trials, not offered clinical trials as an option, or are being treated in community settings where trials may not be available [11]. While some previous studies have examined patient preferences for changes to healthcare delivery systems, there is limited literature incorporating perspectives of women living with MBC, specifically [12].

The goal of this qualitative study was to uncover patients’ ideas for re-imagining care delivery for people living with MBC. In this investigation, we partnered with *Project Life*, an online wellness community founded by those with MBC for those living with MBC, to qualitatively evaluate how members would re-imagine MBC care delivery.

## Methods

Semi-structured interviews were conducted with *Project Life* members, covering individuals’ lived experiences of MBC, greatest unmet needs related to care, and perspectives on virtual wellness community involvement. *Project Life* is a nonprofit, online organization led by individuals living with MBC that freely provides supportive care resources to those with MBC and their caregivers.

### Population

The participants interviewed in this study were members of the *Project Life* virtual wellness community. Purposive sampling was conducted through distributing a study flyer within the *Project Life* community via a closed social media group and an e-mail newsletter. The study flyer included a link to a secure screener survey to assess eligibility. LG, *Project Life* founder and a person living with MBC, distributed this flyer to *Project Life*’s membership of 405 people. The flyer detailed study goals of conducting interviews on the topic of living with MBC and aspects of care delivery. Members were eligible to be interviewed if they identified as living with MBC, were over the age of 18, and could conduct a phone or video interview in English.

### Semi-structured interview development

This entire study design was co-created alongside *Project Life* and *Guiding Researchers and Advocates to Scientific Partnerships (GRASP)* leadership. *GRASP*, which promotes researcher-advocate connections, was included in study design given JM’s role in nurturing the connection between principal investigator, MLR, and *Project Life*’s leadership of LG and DJ. LG, JM, and DJ are all individuals living with MBC and provided direct patient input on the development of the qualitative interview guide.

Co-design included crafting the interview guide through regular biweekly meetings in which the study team identified salient topics in MBC patient communities. Expertise integrated into this process included backgrounds in health services research, medical anthropology, medical oncology, and patient-lived experience. As a result, the interview queried participants’ experiences being diagnosed, and living, with MBC, how care was affected by the COVID-19 pandemic, experiences with cancer care broadly, and feelings about *Project Life* as a virtual wellness community. To gain direct patient insight on how to improve cancer care delivery, participants were asked explicitly “How would you reimagine cancer care delivery for people living with MBC?”.

### Interview conduct and analysis

Once prospective participants were deemed eligible based on screener survey responses, MLR reached out to schedule an interview. Attempt to contact for scheduling was made up to three times. Interviews were conducted virtually over Zoom from March 14, 2022, to May 31, 2022.

All interviews were conducted by MLR, a PhD trained female epidemiologist with a mixed methods certification, and, with informed consent, audio recorded. Recordings were transcribed by *Nvivo* transcription and manually quality checked for accuracy by MLR. Phronetic Iterative Analysis (PIA) was used to systematically analyze the interview transcripts in the mixed-methods software, *MAXQDA* [13]. Incorporating deductive and inductive frameworks and findings, PIA builds a set of salient, inter-related themes through multi-step coding to move data along a spectrum from descriptive to synthetic. Deductive codes were developed through regular meetings with *Project Life* and *GRASP* in addition to a literature review of existing research around patient perspectives on cancer care delivery. Initial, blind coding was conducted by JW and AH. Further non-blind, thematic refinement was performed by MLR. Results were discussed and synthesized with patient stakeholders LG, DJ, and JM, as well as medical oncologist SR for additional contextualization. This study was determined exempt by the Vanderbilt University Medical Center Institutional Review Board (IRB # 220096).

## Results

The study flyer was distributed through the *Project Life* community which included 405 people living with MBC, and from this population, 39 individuals completed the screener survey to assess eligibility. All 39 individuals were deemed eligible for the study. A total of 36 interviews were conducted with women living with MBC who were members *Project Life* (Table 1) from the 39 individuals who completed the eligibility survey. Eligible individuals were contacted up to three times on their preferred mode of communication, phone or e-mail, to schedule the interviews. Three people who completed the screener form were unable to schedule an interview due to non-response. Through the 36 interviews conducted, participants re-imagined the delivery of MBC care in multiple ways. Ideas coalesced around three broad themes: (1) holistic care, (2) information access, and (3) conceptual shifts. Accompanying these themes were several subthemes also discussed below (Table 2).

**Table 1:** Participant demographics (*n* = 36)

Gender	
Women	36 (100%)
Race	
White	28 (77.8%)
Women of color*	8 (22.2%)
Age	
30–45	8 (22.2%)
46–59	15 (41.7%)
60 +	13 (36.1%)
Years living with MBC	
< 5 years	14 (38.9%)
≥ 5 years	21 (58.3%)
Unknown	1 (2.8%)
Introduction source to *Project Life*	
Cancer center	1 (2.8%)
Social media/personal connection	35 (97.2%)
*Project Life* membership length	
< 6 months	4 (11.1%)
6–11 months	9 (25.0%)
12–24 months	19 (52.7%)
Unspecified	2 (5.6%)
Could not remember	2 (5.6%)
Cancer center type (*n* = 48 institutions)	
Academic medical center	21 (43.7%)
Community cancer center	26 (54.2%)
Military hospital	1 (2.1%)

^*^Aggregated due to small sample size. Women of color include women who self-identified as Black, Latina, or Asian

**Table 2:** Themes, subthemes, and illustrative quotations

Theme	Subtheme	Quotes
Holistic care	Organized referrals for non-oncology care	“So I think to re-imagine the side effects. To really, listen to the patients on the side effects. I mean, I am grateful for my doctor because she’s kept the cancer at bay…A lot of doctors try to get you to go back to work when they don’t understand the fatigue level and the impacts of the chemo and the radiation…and neuropathy”
		“And as it relates to therapy, I actually was introduced to the idea by another peer…I never thought about like, hey, this is a trauma. But they talked about how it affects you…and your relationship with yourself, and then how it affects others”
		“They have about six or seven full time social workers and literally one psychiatric nurse for two huge cancer centers”
		“I remember speaking to the social worker once…and I didn’t keep up with that and I didn’t seek out a counselor. And now, like five years later, I’m struggling”
	Caregiver support	“I wish there were something that treated the whole me and my whole family”
	Acceptance of integrative medicine	“Integrative medicine has been an extreme lack in my institution…It wasn’t readily available”
		“They even have acupuncture and…it’s just really, you know, all encompassing care. And I really like that”
Information access	Streamlined clinical trial enrollment	“I think like personalized care like somebody who matches you up with clinical trials and navigates all your insurance for you because. Well, I feel like I’m pretty good with it. There’s so many in the community that are completely lost with all of that stuff, and I think that there’s a lot of opportunity for clinical trials that they have no clue about or the financing behind it”
	Curated high-quality, newly diagnosed patient resources	“So it’s very much I have to find little niches of where to find the appropriate information, which is not easy”
		“But it’s also unfair that I have to find this information myself. So it hasn’t been easy”
Conceptual shifts	MBC-specific terminology and approaches to care	“I still have lots of friends that ask me when my treatments are going to be done you know. I mean, that’s just common. Everybody says that”
	Emphasized long-term life planning and goals-of-care	“If you accept and plan, it frees up your life in a way that you can better enjoy the time you have”
		“I wish things had slowed down a little bit to just say, okay, let’s work through a life plan”
	Centering patient voice and knowledge	“I find it really interesting to hear the science. I had no idea that cancer was this complicated. The genetics, the mutations, it’s just really fascinating”
		“I think we need to be our own best self advocates and see ourselves as partners in our care…with our health care providers”

### Theme 1: Holistic care

While participants felt confidently knowledgeable in receiving direct medical oncology services like chemotherapy and endocrine therapy, they lacked support for medication side effects, mental health needs, integrative treatment options, and caregiver supports. Participants attributed this lack of knowledge to the absence of these elements in clinical conversations with their care teams. One participant summed up this idea succinctly during her interview:

“Everyone should have an oncologist that cares about them as a whole person.”

To overcome barriers to patient-centered metastatic cancer care, patients recommended the following: (a) organized referrals for non-oncology care, (b) caregiver support, and (c) acceptance of integrative medicine.

#### Subtheme: Organized referrals for non-oncology care

Multiple patients expressed frustration that they were not readily connected to non-oncological providers necessary for maintaining well-being throughout MBC treatment. Non-oncology services included referrals to palliative care providers, psychiatrists or counselors, social workers, and patient navigators. As one participant stated:

Well, I think right from the start that more people need to be assigned to each patient, not just a physician and not just a nurse navigator, but you need a psychotherapist. You need nutritional support. You may need…a pulmonary doctor, whatever specialties.

Earlier and more universal referrals to palliative care specialists were especially important to those interviewed.

I think the number one for the majority of people is management of toxicities and side effects that are related to treatment… palliative care, it is not necessarily available everywhere. I have no access to palliative care where I am, and it’s not like I’m in a rural environment. There just aren’t any palliative care doctors…I think that is a big factor in quality-of-life: having access to palliative care…because your oncologist, while they may be great at managing your treatment plan, they’re not necessarily always as knowledgeable as a palliative care doctor can be.

Additionally, participants felt access to psychiatric care, or counseling, would help alleviate some of the mental strain that their metastatic diagnoses and harsh treatments brought.

And as it relates to therapy, I actually was introduced to the idea by another peer…I never thought about like, hey, this is a trauma. But they talked about how it affects you…and your relationship with yourself, and then how it affects others.

I think going through that, the psychiatrist that I talked to was more useful than any of the oncology team…so he was a great resource, but he was hard to get.

Unfortunately, however, participants acknowledged that these providers were scarce, and appointments often resulted in additional travel time.

Despite the importance assigned to them by participants, social workers and other patient navigators, who were cited as helping participants understand insurance issues, connect to support groups, and much more, were frequently cited as overworked and under-resourced. As a participant stated:

Social workers…are the superstars of this program. They are really carrying a lot on their backs and they really know the patients and they really tailor their treatments based on their needs.

Connecting each subtheme concerning organized referrals, participants re-imagined cancer care with less fragmentation to better support MBC patients. Participants imagined not only better care coordination among medical specialties like palliative and psychiatric care, but also among para-medical areas like nutrition, social work, and patient navigation.

#### Subtheme: Caregiver support

Participants recognized the difficulties experienced by caregivers, who were navigating the lived experience of MBC alongside them. For example, one participant emphasized a spouse’s change in employment to better align with her care:

My husband switched to a night job just because of my cancer. Switched careers so that he doesn’t have to take time off. So, you know, we’ll leave at 4:00 in the morning and drive down to do my treatments. And then I usually drive home, so he sleeps and then he works all night.

In addition to the sacrifices made by caregivers, participants also felt that they did not know how to navigate conversations about their diagnosis and future with their loved ones. Multiple patients expressed a desire for care that encompassed not just themselves, but their families as well. Several of the participants were themselves caregivers to young children, ailing spouses, or aging parents. These participants expressed a need for support for their position as caregivers themselves while also navigating living with MBC. Participants with young children sought to re-imagine care delivery in a way that acknowledges and supports their roles as mothers.

I wish that there were more resources out there for people with metastatic cancer that could help them help the people in their life. The thing that took me the longest to check off my list was writing my letter to my daughter. I still cry about it when I think about it because I don’t want to think there’s not a lot of support there for that.

Participants emphasized that the effects of MBC go beyond their individual experience of the disease and have substantial impact on their families and broader support networks. In re-imagining MBC care delivery, participants emphasized acknowledging and supporting those surrounding the patient.

#### Subtheme: Acceptance of integrative medicine

Multiple participants sought candid conversations with their doctors about integrative medicine spanning topics including dietary changes, vitamin supplements, and alternative therapies.

Integrative medicine has been an extreme lack in my institution… It wasn’t readily available.

And then sometimes I wish the conventional medical community would do a better job of acknowledging the complementary piece…I realize there’s both extremes, and I’m not suggesting…saying absolutely no to chemotherapy, and I’m only going to do vitamin C infusion or whatever and cure my cancer that way, I’m not necessarily running out completely to the alternative side, but I really wish there were better ways to blend the two.

However, as demonstrated by the above quotations, participants were not expressing desire for alternative therapies to be truly alternative, so much as they wished for them to be accepted complementary elements to the usual regimen of biomedical care.

### Theme 2: Information access

Participants frequently felt confused about, and frustrated because of, the lack of information available about their diagnosis, prognosis, and treatment options in addition to clinical trial enrollment and metastatic-specific resources. Despite this need, participants felt that physicians did not help them make connections to reliable and easily accessible information, leaving participants feeling solely responsible for their own empowerment through information acquisition.

#### Subtheme: Streamlined clinical trial enrollment

Participants understood the importance of clinical trial enrollment as imperative for advancing the science of cancer care and discovering novel treatments. Several felt that their care teams were not helping them connect to clinical trials or that teams lacked such discussion of clinical trials altogether. As participants noted:

I think [what we need is] personalized care with somebody who matches you up with clinical trials and navigates all your insurance for you because there’s so many in the [MBC] community that are completely lost with all of that stuff, and I think that there’s a lot of opportunity for clinical trials that they [MBC patients] have no clue about or [know] the financing behind.

Well, I think that your oncologist should always have the possibility of clinical trials on the table and be able to articulate what those clinical trials are. And if there doesn’t seem to be one that fits your particular profile, then they will look for one…I think in some of these community hospitals, there’s no discussion of clinical trials.

In turn, participants especially emphasized the need to provide easier access to clinical trials when re-imagining MBC care.

#### Subtheme: Curated high-quality, newly diagnosed patient resources

Websites, journals, and social media groups offer a wide variety of high- and low-quality information to patients. Participants felt challenged when trying to find high-quality, reliable resources about MBC.

Honestly, when I was first diagnosed early stage, I got bamboozled, shall we say, by the natural care world, and I had somebody who is not qualified promise me that I wouldn’t have to do chemo or anything and that she could get me cured, yadda yadda. And I naively believed her because she sounded like she knew what she was talking about, and I knew nothing about the cancer world.

So I think those are the frustrations for me, you know, how do I get information and how do I know what’s good and accurate information?

Participants specifically expressed a desire for curated, evidence-based information from, or endorsed by, their providers or care centers. Participants also emphasized that such resources must be tailored to the complexities of living with metastatic cancer specifically, acknowledging that resources curated for those with early-stage breast cancer are insufficient, and in many cases inappropriate.

### Theme 3: Conceptual shifts

In addition to physically or experientially tangible changes, many participants re-imagined MBC care delivery with paradigmatic shifts in the way providers and the public conceptualize MBC itself. Participants emphasized that living with MBC can drastically alter a patient’s life plans and goals and that physicians and community members must begin to understand what that looks like to provide effective care and support.

#### Subtheme: MBC-specific terminology and approaches to care

Participants felt that, given its incurable nature, the metastatic lived experience is very different from earlier-stage breast cancers. Many participants expressed frustration at mainstream discussions about breast cancer, particularly regarding rhetoric around cure.

[What we need is] Just also having more people understand what the heck MBC is. You know, that’s just as frustrating as all get-out. I still have lots of friends that ask me when my treatments are going to be done you know. I mean, that’s just common.

If [cancer centers] don’t have very many people with metastatic breast cancer and you’re constantly there and you’re in a waiting room with early stage and they’re all doing the pink rah rah ‘B.S.’ and ringing the … bell and all these other things, that can be triggering for people with MBC.

I wish that people would hear, I wish that they would recognize that just because I look good doesn’t mean I am good.

Resultantly, participants felt alienated by comments made by caregivers, friends, and medical professionals and felt that more public education was needed to prevent such misunderstandings.

#### Subtheme: Emphasized long-term life planning and goals-of-care

Participants were keenly aware that while treatments have improved long-term survival for those living with MBC, it remains an incurable disease. During treatment planning and oncology appointments, participants felt that broader discussions about goals-of-care and situating oneself in this new reality were often lost.

How do you reframe your mind to accept that this is reality now, and that kind of goes to the biographical disruption, which for me was a really useful way of talking about it. When you have a terminal disease…You have to reassess so much.

My wanting to ride my bike is part of my life. And it would be nice if the ability to do that was also acknowledged as something important by my medical doctors, you know, my oncologist, I guess.

Overall, participants wanted more of an emphasis on early long-term life planning.

#### Subtheme: Centering patient voices and knowledge

All participants wanted to be welcomed as a member of their care team. With lived experiences and a wealth of knowledge from personal advocacy, patients discussed how they bring their unique expertise into clinical encounters and how it was positively and negatively received.

But we also know a lot about…our disease too, and so I think it’s important to be heard.

When I went to talk with my oncologist about this conversation, she was very dismissive and asked if I had read about it in some blog and didn’t want to hear me, even though I have a master’s degree and I taught research in a local college.

Ultimately, patients felt that the most effective way to re-imagine care in this aspect was for providers to practice a shared decision-making process and patient-centered care.

## Discussion

People living with MBC have undervalued expertise from lived experience that is largely absent from cancer care delivery research. This study partnered with two MBC-patient led organizations to elicit MBC patient perspectives for the improvement of cancer care delivery. Several themes for change were identified, including holistic care, information needs, and conceptual shifts. To achieve these, patients imagined streamlined care with referrals to non-oncological services, caregiver support, acceptance of integrative medicine, MBC-specific terminology and approaches, and centering patient voices throughout.

Aspects of our findings surrounding care communication and coordination are in line with prior qualitative work, examining patient-centered re-design of cancer care that was inclusive of all cancer stages [12]. Our work adds additional nuance focusing on issues that are either more specific or more acute to the metastatic cancer experience such as resource availability. Our work is also in alignment with qualitative research out of Denmark that identified lack of psychosocial support as a major barrier for patients with MBC [14]. A study using qualitative social media data further identified several overlapping issues including palliative care, patient navigation, and clinical trials access [15]. There has been extensive prior work conducted on advanced care planning and receipt of palliative services among individuals with metastatic cancers [16–20]. In addition, prior literature has emphasized caregiver support as a priority area in oncology, particularly for those with metastatic cancers [6, 8, 21–24]. Put together, our work as well as the work of several others directly expands the patient voice in the re-imagining of cancer care delivery to better serve individuals with metastatic breast cancer [12, 14, 15].

The emphasis on integrative and complementary medicine in our study potentially represents the burgeoning interest in supportive care outside of traditional Western clinical norms. Globally, integrative medicine has a centuries long history that has slowly made its way into the contemporary USA [25]. As a result of this growing interest, in 2021, the American Society for Clinical Oncology in partnership with the Society for Integrative Oncology issued a joint guideline for the use of integrative medicine, like acupuncture, for pain management in cancer patients [26]. The development of this guideline represents a notable shift in the acceptance of integrative medicine into the Western clinical mainstream. Beyond pain management, integrative therapies have also been used to manage breathlessness in advanced cancer patients [27]. In addition, mindfulness-based interventions are gaining traction for supportive care to supportive individuals with metastatic cancer [28–31]. Like many aspects of cancer care, insurance coverage for integrative therapies remains patchy, potentially resulting in cost burden to patients [32]. While several participants in our study had positive experiences speaking with their care teams about integrative medicine, several felt shut down around these conversations. Given the immense symptom burden often associated with MBC, providing well-vetted integrative medicine resources to interested patients is paramount.

This study has several notable strengths. This investigation was co-created with people living with MBC, thus centering the patient voice in the study design, conduct, and analysis. Participants spanned age ranges, racial groups, time living with MBC, and care setting. Given that most cancer health services research focuses on patient populations treated at academic medical centers, this diversity of experience added great breadth to our findings. However, this study is not without limitations. We were able to interview individuals who were physically well enough to participate in the study, meaning the perspectives of patients with the highest medical need were not included. Furthermore, we intentionally focused on people living with MBC, but metastatic disease affects more than just the individual, and as such, future research should be inclusive of caregiver perspectives.

## Conclusion

In conclusion, people living with MBC have myriad perspectives on how to improve cancer care delivery grounded in lived experience. Study participants emphasized the need for more integrated care, ease of information access, and more MBC-specific resources. To truly re-imagine MBC care delivery, patient voices must be centered in care innovation.

## Data Availability

The datasets generated during the current study are not publicly available because of the potential for individual participant privacy and confidentiality to be compromised.
